# Radiosensitizing Effects of Irinotecan versus Oxaliplatin Alone and in Combination with 5-Fluorouracil on Human Colorectal Cancer Cells

**DOI:** 10.3390/ijms241210385

**Published:** 2023-06-20

**Authors:** Bernd Frerker, Felix Bock, Marie-Louise Cappel, Stephan Kriesen, Gunther Klautke, Guido Hildebrandt, Katrin Manda

**Affiliations:** 1 Department of Radiotherapy and Radiation Oncology, University Medical Center Rostock, Suedring 75, 18059 Rostock, Germany; bernd.frerker@med.uni-rostock.de (B.F.); felix.bock@med.uni-rostock.de (F.B.); maloucappel@gmail.com (M.-L.C.); stephan.kriesen@med.uni-rostock.de (S.K.); guido.hildebrandt@uni-rostock.de (G.H.); 2 Department of Radiation Oncology, Hospital Chemnitz, Bürgerstrasse 2, 09113 Chemnitz, Germany; g.klautke@skc.de

**Keywords:** irinotecan, oxaliplatin, 5-fluorouracil, ionizing radiation, radiosensitizer, HT-29, cancer cells

## Abstract

To date, oxaliplatin and irinotecan are used in combination with 5-flourouracil (5-FU) for metastatic colorectal cancer. In this study it was tested whether oxaliplatin and irinotecan and their combinations with 5-FU have an enhanced effect when treated simultaneously with ionizing radiation. In addition, it should be compared whether one combination therapy is more effective than the other. Colorectal cancer cells (HT-29) were treated with irinotecan or oxaliplatin, both alone and in combination with 5-FU, and subsequently irradiated. The cell growth, metabolic activity and proliferation of cells were investigated, and the clonogenic survival was determined. Furthermore, the assessment of radiation-induced DNA damage and the influence of the drugs and their combinations on DNA damage repair was investigated. Treatment with irinotecan or oxaliplatin in combination with 5-FU inhibited proliferation and metabolic activity as well as clonogenic survival and the DNA damage repair capacity of the tumor cells. The comparison of oxaliplatin and irinotecan with simultaneous irradiation showed the same effect of both drugs. When oxaliplatin or irinotecan was combined with 5-FU, tumor cell survival was significantly lower than with monotherapy; however, there was no superiority of either combination regimen. Our results have shown that the combination of 5-FU and irinotecan is as effective as the combination of 5-FU with oxaliplatin. Therefore, our data support the use of FOLFIRI as a radiosensitizer.

## 1. Introduction

Stage II and III rectal cancer is traditionally treated with a multimodal approach consisting of neoadjuvant 5-fluorouracil (5-FU)-based chemoradiation, followed by a surgery and adjuvant chemotherapy [1]. It is superior to perioperative radiotherapy alone [2,3,4], and continuous 5-FU infusion has been shown to be more effective than a bolus [5]. Compared with adjuvant irradiation, local control rates (LCR) are improved, but there is no effect on overall survival (OS) or disease-free survival (DFS), respectively [6]. To improve these endpoints, several trials have changed the timing and sequence of the three treatment modalities or incorporated new agents such as oxaliplatin or irinotecan into the treatment regimens. Recently, in 2021, the results of the RADIPO [7] and PRODIGE 23 [8] phase 3 trials have been published. These trials investigated a total neoadjuvant therapy (TNT) approach in which the chemotherapy, consisting of 5-FU, leucovorin and oxaliplatin (FOLFOX, [7]) or additionally irinotecan (FOLFIRINOX, [8]), was administered as induction or consolidative chemotherapy before or after the irradiation, but not concurrently. Subsequently, the surgery was performed. TNT significantly improved DFS [7,8], and it became a new standard of care. In addition to TNT, oxaliplatin has been introduced as a radiosensitizer [9] in clinical trials, and, subsequently, several randomized phase 3 trials investigated the effect of adding oxaliplatin to 5-FU based chemoradiation [10,11,12,13,14,15,16]. DFS was significantly better in the CAO/ARO/AIO-04 trial [10], but little effect was found in the other trials [11,12,13,14,15,16]. Similarly, irinotecan has been used as a radiosensitizer [17] alone or in combination with 5-FU in several phase 2 trials [18,19,20,21,22,23,24,25,26,27,28,29,30,31,32,33], but only a few phase 3 trials have been reported [34,35]. The current CAO/ARO/AIO-18-trial investigates the addition of oxaliplatin to a 5-FU based chemoradiation as part of the TNT, but the results are not yet published. That is why the administration of oxaliplatin and irinotecan concomitant to chemoradiation is not currently indicated in national guidelines [36,37,38].

Nevertheless, preclinical data [9,17] support the use of oxaliplatin and irinotecan as radiosensitizers [39,40,41,42]. The radiosensitive activity of oxaliplatin was first described by Blackstock et al. in 1999 [43] in an abstract of the 41st annual ASTRO meeting, and, in the last two decades that followed, several preclinical studies investigated its cytotoxic effects in combination with irradiation but not on human colorectal cancer cells [44,45,46,47,48,49,50,51,52,53,54,55,56,57,58]. Similar, preclinical studies reported on the cytotoxic effects of irinotecan [17] on colorectal cancer [59,60,61,62,63,64,65,66,67] or non-colorectal [68,69,70,71,72,73,74,75,76] cancer cells. The aim of these studies was to determine the influence of oxaliplatin [44,46,47,51,52,53,54,55,57] and irinotecan [63,64,65,66] on cellular pathways, cell cycle and mechanisms of drug resistance or to describe mechanisms of radiosensitization and radioresistance with oxaliplatin [45,48,49,50,56,58] and irinotecan [59,60,61,62,67]. Other preclinical studies compared the effects of oxaliplatin with those of irinotecan [77,78,79,80,81,82,83,84,85,86,87], but, to our knowledge, there are very few data that have compared in detail the radiosensitizing effects of adding oxaliplatin or irinotecan to 5-FU based chemoradiation (FOLFOX or FOLFIRI, respectively) on human colorectal cells in a preclinical model. The possible underlying pathophysiological mechanisms are poorly described in the literature. Thus, the aim of this study was to investigate and to compare the radiosensitizing effects of oxaliplatin and irinotecan with or without 5-FU at the cellular level using in vitro studies on human HT-29 colorectal cancer cells.

## 2. Results

### 2.1. Cell Growth

**Figure 1:**
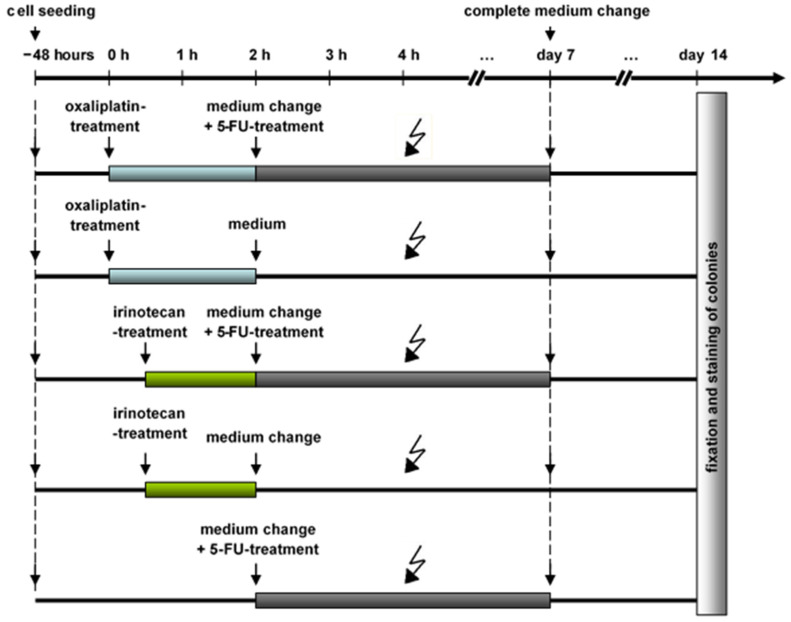
Schematic representation of the colony formation test experimental setup with the active ingredient regimens used, as well as the time of seeding, irradiation, medium change and evaluation, with addition and incubation times also. 5-FU: 5-flourouracil.

To investigate the influence of all three drugs on the growth of the HT-29 human colorectal tumor cells, the three drugs (irinotecan, oxaliplatin and 5-FU) were tested separately. In accordance with clinical practice, a change of medium occurred after 2 h for oxaliplatin and 1.5 h for irinotecan, and no change occurred for 5-FU (Figure 1).

**Figure 2:**
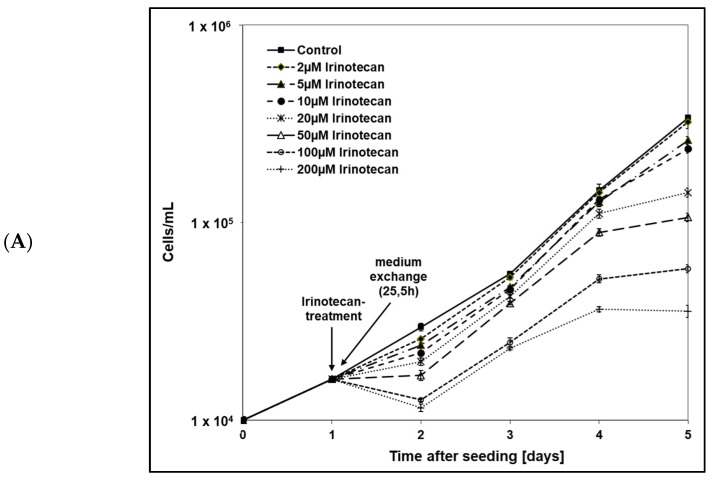

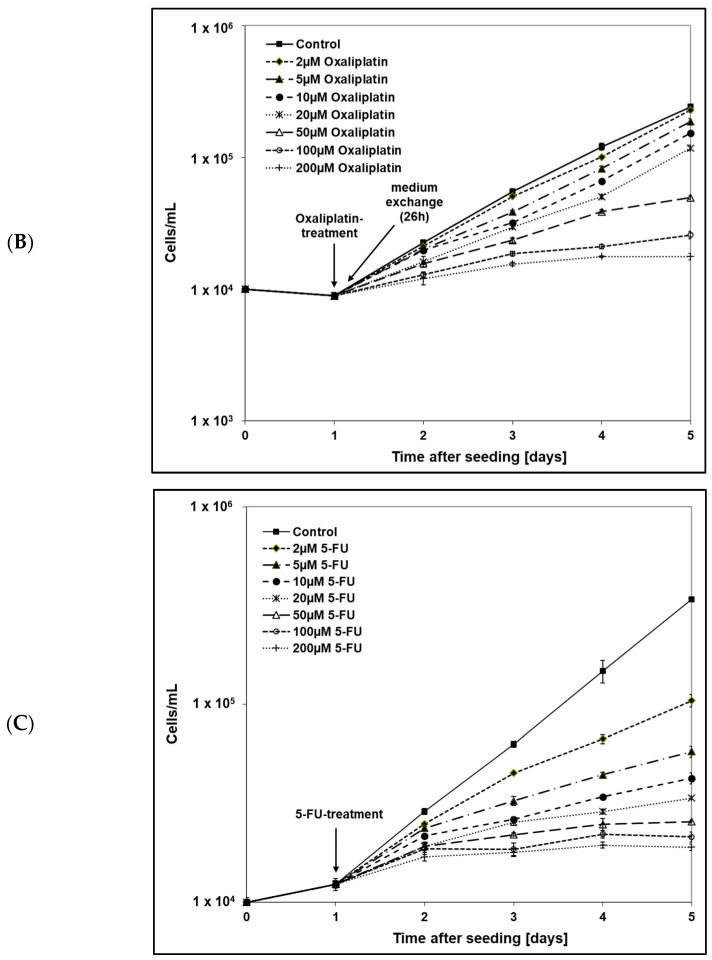
Inhibitory effect of increasing concentrations of (**A**) irinotecan, (**B**) oxaliplatin and (**C**) 5-FU on cell growth of HT-29 cells. The cells were seeded on day 0. Drugs were added 24 h later with various concentrations. Medium change was performed 1.5 h (irinotecan), 2 h (oxaliplatin) or not at all (5-FU) after drug incubation. Error bars indicate the standard deviation for three independent experiments; wells were assayed in triplicate in each of the different experiments.

Increasing the concentrations of all three drugs reduced the growth of the human tumor cells (Figure 2).

The cytotoxic effect was concentration-dependent, but apparently the sensitivity of the cell line to these three drugs was different. While 24 h after treatment the growth of HT-29 cells was clearly inhibited by irinotecan, the growth inhibition by oxaliplatin and 5-FU was not as strong. At later time points during the incubation time, the effect of 5-fluorouracil was more pronounced than any of the other two drugs. IC_50_ values were calculated at day 5 (after a four-day drug incubation time) and were 19.02 µM (Oxaliplatin), 17.06 µM (Irinotecan) and 1.44 µM (5-FU). Due to the results of growth curves, oxaliplatin and irinotecan were used at concentrations of 20 µM for the subsequent experiments. The lowest concentration of 5-FU showed a significant inhibition of cell growth, so subsequent experiments were performed with a 5-FU concentration of 2 µM.

### 2.2. Clonogenic Survival

**Figure 3:**
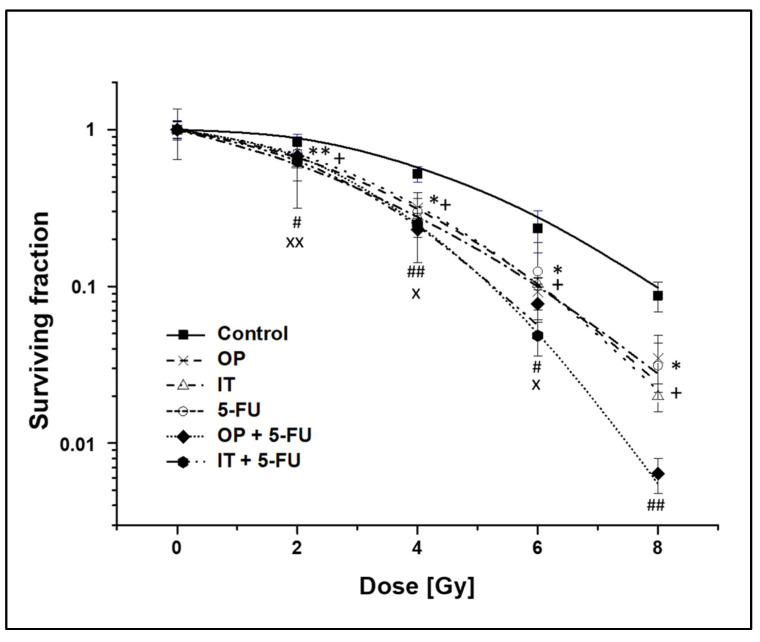
Clonogenic survival of HT-29 cells pre-treated with irinotecan (IT) vs. oxaliplatin (OP) alone or in combination with 5-fluorouracil (5-FU) before irradiation. Clonogenic fractions of treated cells were normalized to the plating efficiency of untreated controls. Error bars indicate the standard errors for three independent experiments; flasks were assayed in duplicate in each of the different experiments. Asterisks illustrate significance: *^/+^ *p* < 0.05, ** *p* < 0.01 for OP (*) and IT (^+^) related to the control of the respective radiation dose; ^#/x^ *p* < 0.05, ^##/xx^ *p* < 0.01 for OP (^#^) and IT (^x^) related to the treatment with vs. without 5-FU of the respective radiation dose.

To investigate the potential role and compare the treatment regimens of irinotecan vs. oxaliplatin alone and in combination with 5-fluoruracil as radiosensitizers, the colony-forming ability was determined. For the combined drug pretreatment before irradiation, a specific time schedule, according to clinical administration, was used (Figure 1). The survival of the tumor cells was reduced with an increasing of the radiation doses (Figure 3).

The substances alone (oxaliplatin, irinotecan, 5-FU) each led to a significant increase in the radiation response of the tumor cells. In addition, the radiation sensitivity of the tumor cells was further significantly increased with the combination of oxaliplatin or irinotecan with 5-FU. The therapy regimens (oxaliplatin + 5-FU vs. irinotecan + 5-FU) did not differ significantly from each other with regard to their radiation-sensitizing effect.

### 2.3. DNA DSB Repair Capacity

**Figure 4:**
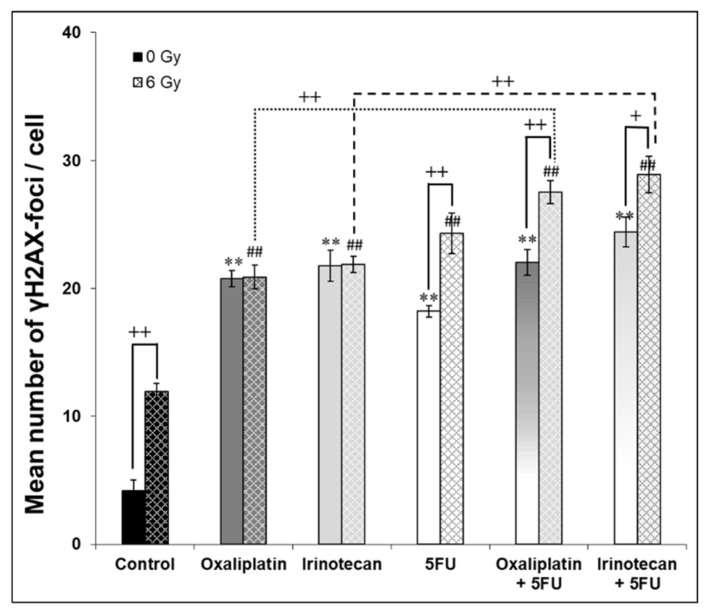
Effect of irinotecan (20 µM) vs. oxaliplatin (20 µM) alone and in combination with 5-fluorouracil on DNA DSB repair capacity 24 h after irradiation. Error bars indicate the standard deviation for three independent experiments. Asterisks illustrate significances: ** *p* ≤ 0.01 related to the untreated control (0 µM, 0 Gy); ^##^ *p* ≤ 0.01 related to the irradiated control (0 µM, 6 Gy), or ^+^ *p* ≤ 0.05, ^++^ *p* ≤ 0.01 related as shown with bars.

The purpose of this test was to investigate whether there was a significant difference between the different drug regimens in terms of the number of DNA double-strand breaks. At the same time, it was of interest whether the repair capacity of the cells is impaired after damage from ionizing radiation and whether there is a difference between the irradiated and the non-irradiated approaches. The greatest difference in the number of DNA double-strand breaks between the irradiated and non-irradiated batches was found for the control cells treated without an active substance (Figure 4).

However, the cells under the influence of 5-FU and under the drug combinations (oxaliplatin + 5-FU and irinotecan + 5-FU) also showed a significantly reduced repair capacity of the radiation-induced DNA damage. The combination of oxaliplatin or irinotecan with 5-FU was more effective than the single agent administration. Once again, there was no significant difference between the two therapy regimens oxaliplatin + 5-FU and irinotecan + 5-FU for DNA DSB repair capacity.

### 2.4. Metabolic Activity

**Figure 5:**
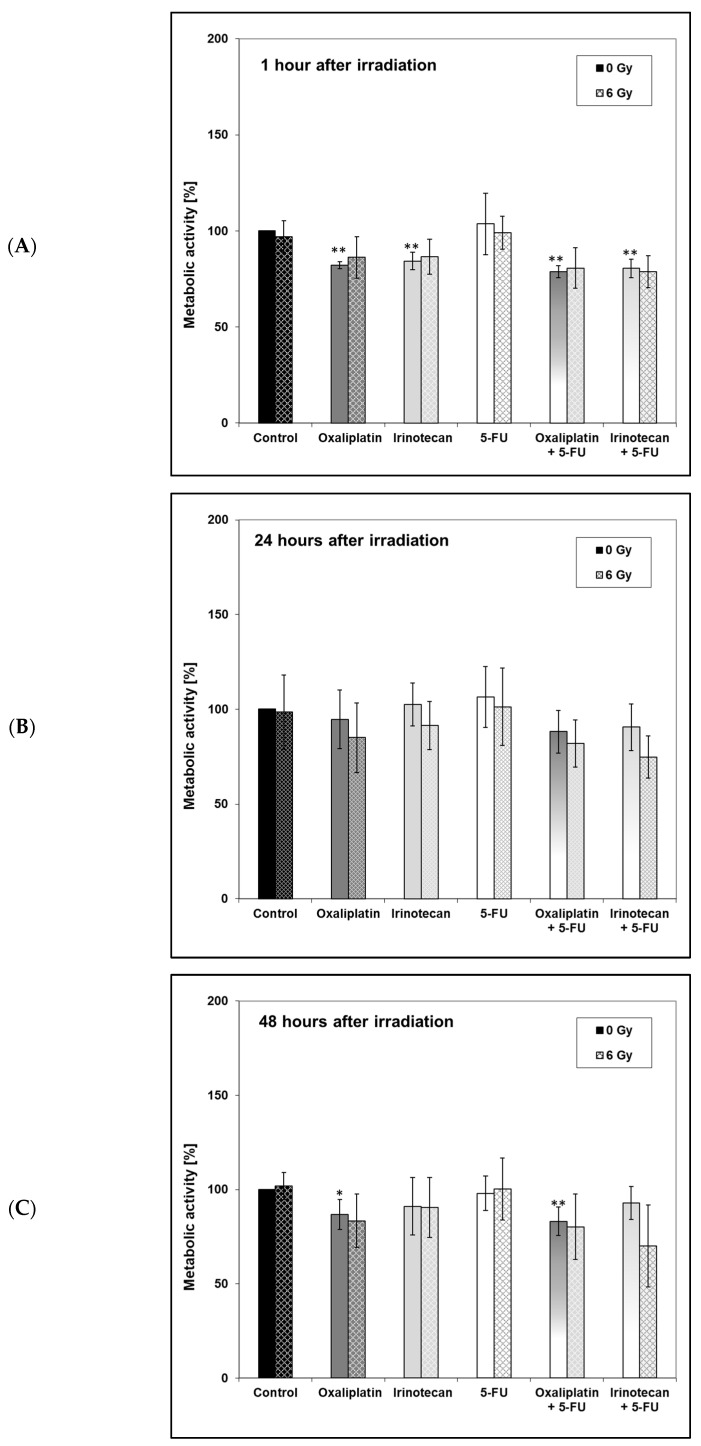
Metabolic activity of HT-29 cells (**A**) 1 h, (**B**) 24 h or (**C**) 48 h after irradiation under treatment with irinotecan vs. oxaliplatin alone and in combination with 5-fluoruracil. Drugs were added 4 h (oxaliplatin; 20 µM) or 3.5 h (irinotecan; 20 µM) before irradiation. At 2 h before irradiation, a complete medium change was performed. For irinotecan vs. oxaliplatin experiments, medium without any drug was added; for experiments in combination, 5-fluoruracil (5-FU; 2 µM) was added. Irradiation followed 28 h after cell seeding. Error bars indicate the standard deviation for five independent experiments; wells were assayed in triplicate in each of the different experiments. Asterisks illustrate significances: * *p* ≤ 0.05, ** *p* ≤ 0.01 related to the untreated control (0 µM, 0 Gy).

First, the metabolic activity of the HT-29 cells was analyzed 1 h after irradiation. When unirradiated cells were treated with oxaliplatin or irinotecan alone or in combination with 5-FU, the metabolic activity was significantly lower than that of the unirradiated control (Figure 5A). No significant effect of metabolic activity at 6 Gy compared to metabolic activity at 0 Gy was demonstrated for any of the drug regimens examined.

At 24 h, there was no difference between the unirradiated control and all unirradiated drug-treated cells (Figure 5B). The metabolic activity for all irradiated experimental batches, including the control, was below the value determined at 0 Gy. Again, significant differences could not be demonstrated with 6 Gy compared to 0 Gy.

Finally, the metabolic activity 48 h (Figure 5C) after the irradiation time point was highest under the influence of 5-FU. Compared to the untreated control (0 µM, 0 Gy) significant differences in the metabolic activity of the cells for the unirradiated batches under the influence of oxaliplatin (*p* < 0.05) and oxaliplatin + 5-FU (*p* < 0.01) were detectable.

Overall, there was no significant difference in metabolic activity between the two drug regimens oxaliplatin + 5-FU and irinotecan + 5-FU at any of the three measurement times when the cells were irradiated at the same time.

### 2.5. Proliferation

**Figure 6:**
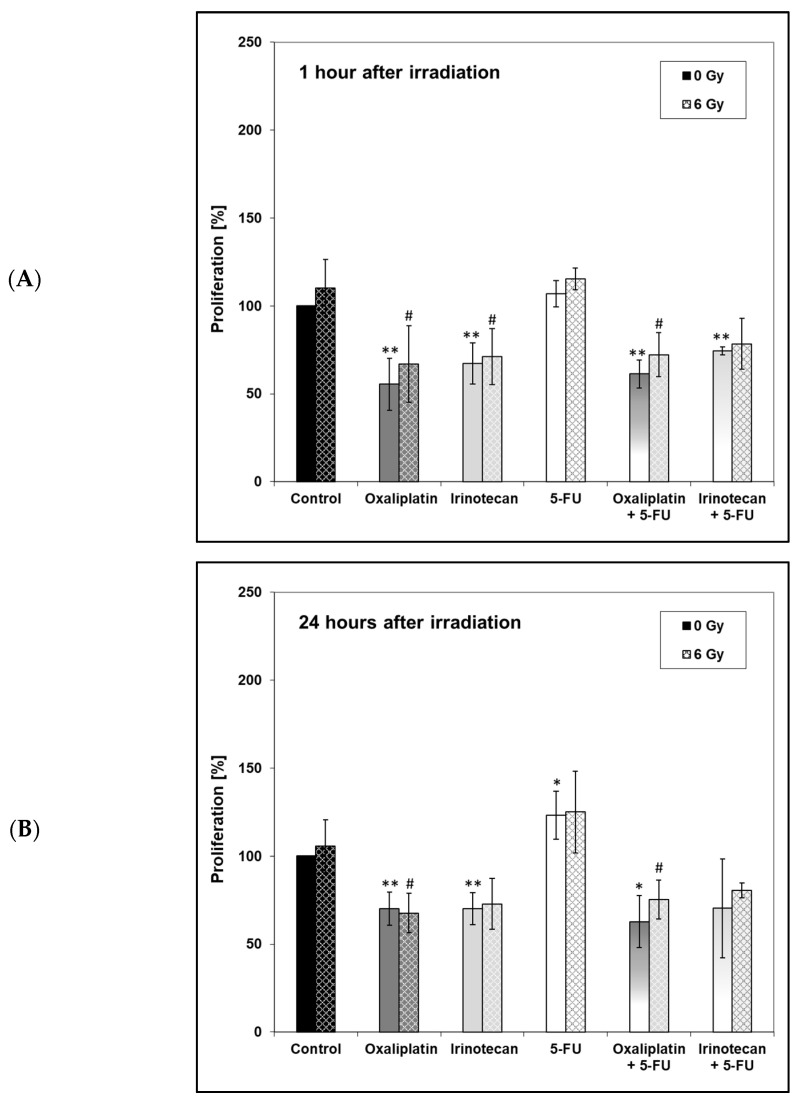

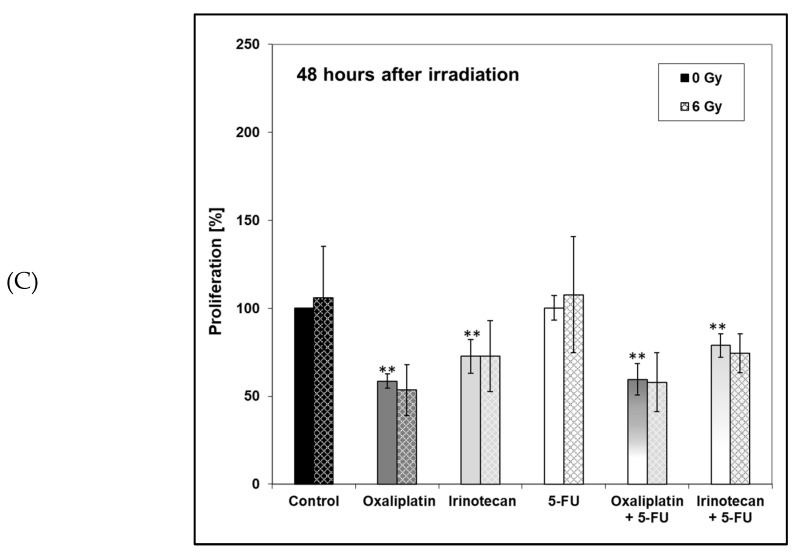
Proliferation of HT-29 cells after incubation with different drugs (**A**) 1 h, (**B**) 24 h or (**C**) 48 h after irradiation. Drugs were added 4 h (oxaliplatin) or 3.5 h (irinotecan) before irradiation. At 2 h before irradiation a complete medium change was performed. For irinotecan vs. oxaliplatin experiments, medium without any drug was added; for experiments in combination, 5-fluoruracil (5-FU) was added. Irradiation followed 28 h after cell seeding. BrdU incubation time 1.5 h; untreated controls = 100%. Error bars indicate the standard deviation for three independent experiments; wells were assayed in eight replicates in each of the different experiments. Asterisks illustrate significances: * *p* ≤ 0.05, ** *p* ≤ 0.01 related to the untreated control (0 µM, 0 Gy); ^#^ *p* ≤ 0.05 related to the irradiated control (0 µM, 6 Gy).

Proliferation of the HT-29 cells was also analyzed 1 h, 24 h and 48 h after the time of irradiation (Figure 6).

At all three time points, in comparison with the untreated control, the proliferation was inhibited when the unirradiated cells were treated with oxaliplatin or irinotecan alone or in combination with 5-FU. However, overall, similar to the metabolic activity, no significant differences between the two drug regimens, oxaliplatin + 5-FU or irinotecan + 5-FU, could be observed at any of the three measurement times.

## 3. Discussion

The incorporation of new agents such as oxaliplatin or irinotecan into treatment regimens for colorectal cancer is a promising approach to improve the efficacy of therapy, but there are still few data directly comparing the radiosensitizing effects of the drug combination of oxaliplatin and 5-FU (FOLFOX) with those of irinotecan and 5-FU (FOLFIRI). Therefore, we aimed to investigate the radiosensitizing effects of oxaliplatin or irinotecan alone and their combinations with 5-FU on the cellular level. Our major finding is that the combination of oxaliplatin + 5-FU is as effective as the combination of irinotecan + 5-FU.

Firstly, we investigated the chemosensitivity of each single drug without irradiation to determine the drug concentrations we used for subsequent experiments. Cell growth of HT-29 cells was inhibited in a concentration-dependent manner after administration of oxaliplatin, irinotecan, and 5-FU alone. In previous studies, the concentration-dependent decrease in cell growth and various corresponding IC_50_ values have been reported for HT-29 cells [53,55,61,65,85] and similarly for other colorectal cells [44,46,51,57,65,88,89]. The reported IC_50_ values differ among the studies due to differences in the incubation time and the allowed duration of cell growth following drug incubation. In our study, the calculated IC_50_ values resulted in a concentration of approximately 20 µM for oxaliplatin and irinotecan and 2 µM for 5-FU. Thus, our results confirm the cytotoxicity of the three drugs we used.

We then examined the radiosensitizing effect of the three active agents alone or in combination with respect to the colony forming ability. Even without irradiation, it was previously reported that the colony forming ability in colorectal cancer cells was reduced after incubation with oxaliplatin [88], irinotecan [90] or 5-FU [89]. Our data confirm these observations. With irradiation, the colony forming assay showed a significant reduction in clonogenic survival with an increasing irradiation dose for each drug alone. This effect was further enhanced when oxaliplatin or irinotecan were used in combination with 5-FU. In other studies, the radiosensitizing effects of the three agents were investigated. Folkvord et al. [50] incubated HT29 and HCT116 with oxaliplatin (immediately before irradiation for 2 h) or 5-FU (immediately after irradiation for 48 h), and 10 days later, colonies were counted. The increasing doses of irradiation reduced survival and the addition of 5-FU or oxaliplatin enhanced the effect. Similar to our results, the combination of oxaliplatin, 5-FU and irradiation was the most effective. Regarding irinotecan, similar results were obtained. Wang et al. [61] incubated HT-29 cells with irinotecan for 24 h, and the clonogenic assays was performed 10 days later. Omura et al. [67] investigated the effect of SN-38 (7-ethyl-10-hydroxycamptothecin), a metabolite of irinotecan on HT29. Cells were treated with SN-38, immediately irradiated and incubated for an additional 4 h. Then, 12 days later, the colonies were counted. Throughout both studies, with an increasing concentration of irinotecan and an increasing irradiation dose, the ability to form colonies decreased. It is noteworthy that SN-38 significantly enhanced the cytotoxic effect of irradiation in cells grown as spheroids, whereas in monolayers, the effect was marginally enhanced. In addition, some studies compared the effects of oxaliplatin and irinotecan. In the study by Shelton et al. [81], HT29 and HCT116 cells were treated for 2 h with oxaliplatin, irinotecan or 5-FU; irradiated; and allowed to grow for 10 days. The colony-forming ability was reduced with increasing irradiation doses, and these results were similar to those obtained by Frey et al. [83]. However, in the latter two studies, the active agents were not combined. In summary, previously published studies [50,56,61,67,81,83] described the radiosensitizing effect of oxaliplatin, irinotecan and 5-FU in the colony formation assay. The strength of our study is that we directly compared the radiosensitivity of oxaliplatin and irinotecan with or without 5-FU. We found that the combinations of oxaliplatin + 5-FU or irinotecan + 5-FU were significantly more radiosensitive than the treatments with oxaliplatin or irinotecan alone. Furthermore, we have shown that the combination of oxaliplatin + 5-FU is as effective as irinotecan + 5-FU.

The success with any of the drug or irradiation experiments were performed to identify possible cellular mechanisms to explain the observed effects in the colony formation assay. First, we analyzed the DNA repair capacity using the γH2AX immunostaining. It is well known that irradiation introduces DNA double strand breaks [91]. A suitable marker for the DNA damage is the immunostaining of the phosphorylation at serine 139 of the histone H2AX after irradiation [92,93,94]. Compared with the corresponding control, treatment significantly induced more γH2AX foci. This was previously reported by Wang et al. [61]. They found the most γH2AX foci in cells treated with irinotecan and irradiation at the same time. In addition, Park et al. [80] reported an increase of γH2AX foci following a single treatment with oxaliplatin, irinotecan or irradiation. However, in our study, cells treated with 5-FU or the drug combinations (oxaliplatin + 5-FU and irinotecan + 5-FU) showed a significantly impaired repair capacity of the radiation-induced DNA damage after 24 h. Moreover, as already observed in the colony forming, the combination of oxaliplatin or irinotecan with 5-FU was more effective than the single agent administration. Once again, there was no significant difference between the two therapy regimens oxaliplatin + 5-FU and irinotecan + 5-FU for DNA DSB repair capacity. Thus, the damage to the DNA might result in reduced cell survival as observed in the colony-forming ability. This strongly suggests that FOLFOX or FOLFIRI can be used as an effective radiosensitizer, and oxaliplatin + 5-FU is as effective as irinotecan + 5-FU.

Besides the DNA repair capacity, we analyzed the metabolic activity (as measured with the XTT-assay) and cell proliferation (as measured with the BrdU-assay). In our study, metabolic activity was significantly lower in unirradiated cells treated with oxaliplatin or irinotecan alone or in combination with 5-FU (1 hour afterwards) or treated with oxaliplatin and its combination with 5-FU (48 h afterward) compared to the unirradiated control. There were no significant differences between the irradiated control and the irradiated drug-treated cells. In addition, no significant effect of metabolic activity at 6 Gy compared to metabolic activity at 0 Gy was demonstrated for any of the drug regimens examined. In the study by Xu et al. [95], there was also no difference between oxaliplatin- and 5-FU-treated SW837 cells, but the combination of both drugs significantly reduced the metabolic activity in irradiated cells. This is different from our study and might be due to different colorectal cell lines. Magné et al. [58] examined ionizing irradiation and its combination with 5-FU and oxaliplatin on two human colorectal cancer cells, WiDr (p53 mutated, mut) and SW403 (wild type, wt). Oxaliplatin sensitized both cell lines to irradiation, whereas 5-FU only sensitized SW403 but not WiDr. Similarly, when oxaliplatin and 5-FU were applicated at the same time, a synergistic interaction between irradiation and drug application occurred in SW403 (p53wt) cells but not in WiDr (p53mut) cells. It was concluded that the p53 status influenced radiosensitivity. The HT-29 chells we used are also characterized by a p53 mutation. Since we found no effect in irradiated cells treated with oxaliplatin and 5-FU, our results correspond to those of Magné et al. With regard to irinotecan, Ebrahimpour [59] demonstrated a significant reduction in the MTT-assay in HCT-116 cells 24 h after the treatment with irradiation or irinotecan. In our study, there was no difference at this time point, possibly due to different cell lines. In addition, a reduced metabolic activity after treatment with oxaliplatin, 5-FU and their combination was described by Fischel et al. [86], but they did not investigate the effect of irradiation. A study by Kjellström et al. [56] also aimed to compare the antitumoral effects of 5-FU and oxaliplatin with irradiation on the human colorectal cancer cell line S1 via MTT-assay. Cells were incubated for 2 h with oxaliplatin and afterwards treated with a short-term (1 h, bolus) or a long-term (24 h, continuously) exposure of 5-FU. Irradiation followed at the end of the drug application or 1 h after the beginning of the long-term exposure of 5-FU. Seven days later, the MTT-test was performed. Increasing radiation doses reduced metabolic activity, and the addition of 5-FU or oxaliplatin enhanced the effect. The results of our proliferation assay were similar to the metabolic assay. Interestingly, 1 h and 24 h after irradiation, there was a significant difference between the irradiated control and cells that were treated with irradiation and oxaliplatin + 5-FU. However, our results are indicating that the observed effect in the colony-forming ability may not be explained by an impaired metabolic activity or by cell proliferation.

Some limitations apply to our study.

First, we performed the experiments after the colony formation test with only 6 Gy. Conventional single doses of 2 Gy are often used in the clinic, and the radiogenic toxicity correlates with the cumulative dose. These effects cannot be directly translated to preclinical models. Wang et al. [61] stated that, at lower radiation doses (2 Gy or 4 Gy), the results of the experiments were inconclusive, and higher doses (> 6 Gy) resulted in severe toxicity, confounding the results. In our colony-forming ability, the results were more obvious after irradiation with 6 Gy or more Gy. Therefore, we used 6 Gy for the subsequent experiments.

Second, we did not test for apoptosis. This has been described extensively in the literature: Arnould et al. [85] performed extensive experiments to analyze apoptosis of colorectal cells after treatment with irinotecan and oxaliplatin, and the study by Frey et al. [83] examined the effects of irradiation on cells treated with oxaliplatin, irinotecan and 5-FU.

Third, we did not use folinic acid. In the clinical setting, folinic acid is added to 5-FU [96,97,98]. However, as stated by Rödel et al. [5], folinic acid does not necessarily have a synergistic effect, and in the study by Kjellström, folinic acid was omitted [56]. Therefore, we also decided not to use folinic acid in our experiments—also to avoid other influencing factors.

Fourth, we only investigated HT29 cells. This cell line expresses a mutation in the p53 gene. In the study by Magné et al. [58], oxaliplatin had no effect on WiDr cells, a cell line that expresses a p53 mutation. In contrast, Folkvord et al. [50] found similar effects of oxaliplatin on both p53 mutated (HT29) and p53 wildtype (HT116) cells. Therefore, the p53 status should not necessarily have an effect on the oxaliplatin-induced radiosensitization [99].

Finally, we did not test several sequences and timing schedules of the drug application and its combination with irradiation. The importance of the optimal treatment plan has been described several times in the literature [100,101,102,103,104]. However, an optimal schedule was proposed by Kjellström et al. [56], and our results fit well with the proposed schedule.

In conclusion, we could show that after the combination of oxaliplatin or irinotecan with 5-FU, the cells were more radiosensitive than after the monotherapy with oxaliplatin or irinotecan alone. Furthermore, we have proven that oxaliplatin + 5-FU is as effective as the combination of irinotecan + 5-FU. The colony forming ability was clearly impaired, which was, possibly, mainly due to the damaged DNA rather than an impaired metabolic activity or cell proliferation. Our preclinical results are in line with the RTOG 0247 phase II trial [105,106] that compared the neoadjuvant radiotherapy with capecitabine and oxaliplatin—respectively, capecitabine and irinotecan. The pathological complete response was found to be better for oxaliplatin/capecitabine [105]; OS, LC and DFS were similar [106], but, to our knowledge, no phase III trial followed. In summary, our preclinical results confirm the use of irinotecan in combination with 5-FU as a radiosensitizer. Irinotecan may be a reasonable alternative to oxaliplatin in patients who have contraindications to oxaliplatin. Irinotecan in combination with 5-FU concurrent with radiotherapy should be further evaluated in phase 3 trials.

## 4. Materials and Methods

### 4.1. Cell Culture

The human colorectal cancer cell line HT-29, obtained from DSMZ (German Collection of Microorganisms and Cell Cultures, Braunschweig, Germany), was cultivated with Dulbecco’s Modified Eagle Medium (DMEM; Biochrom AG, Berlin, Germany) supplemented with 10% fetal bovine serum (FBS) and 1% penicillin/streptomycin (both PAA Laboratories GmbH, Cölbe, Germany) at 37 °C and 5% CO_2_ humidified atmosphere. The cells used in the experiments were passaged twice weekly to ensure exponential growth and grown as epithelial monolayers in tissue culture flasks. A confluence of less than 80% was used to avoid multilayer cell growth.

### 4.2. Drug Preparation

Oxaliplatin, irinotecan-monohydrochloride and 5-Fluorouracil (5-FU) were purchased from Sigma-Aldrich Chemie GmbH (Steinheim, Germany) and dissolved in Aqua ad iniectabilia (B. Braun Melsungen AG, Melsungen, Germany) to generate a 5 mM stock solution of each drug. DMEM was used for further dilutions. The stock solutions were stored in aliquots at −18 °C. If not otherwise mentioned, cells were incubated with oxaliplatin for 2 h [96,107] and with irinotecan for 1.5 h [97,108] before irradiation or continuously with 5-Fu [1] according to clinical practice.

### 4.3. Cell Growth under Drug Treatment

The HT-29 cancer cells were seeded into 24-well plates at a density of 1 × 10^4^ cells per well. Cells were treated after attaching for 24 h with 5-FU, oxaliplatin or irinotecan, respectively, with increasing concentrations (0 µM control; 2 µM, 5 µM, 10 µM, 20 µM, 50 µM, 100 µM and 200 µM). Following drug incubation, medium changes were performed 1.5 h (irinotecan), 2 h (oxaliplatin) or not (5-FU) after cell treatment. Every day after 24 h, the cells were trypsinzed, and the cell number was counted with a Coulter Counter (Z2, Beckman Coulter GmbH, Krefeld, Germany). Each concentration value and the controls were determined in triplicates, and experiments were run over 5 days (120 h). Altogether, three independent experiments were performed.

### 4.4. Irradiation

Irradiation of cells was administered at room temperature utilizing a Siemens Oncor expression linear accelerator (Siemens, Erlangen, Germany) at a dose rate of 3.75 Gy/min (energy 6 MeV). The irradiation doses used were 0 Gy (control), 2 Gy, 4 Gy, 6 Gy and 8 Gy (each single fraction).

### 4.5. Colony-Forming Assay

Clonogenic cell survival was determined with the colony-forming assay. Cancer cells were plated in T25-flasks at a density of 1.5 × 10^3^ cells and were allowed to growth for 48 h. Drug treatment was given 4 h (oxaliplatin, 20 µM) or 3.5 h (irinotecan, 20 µM) before irradiation. Schematic representation of the colony formation test experimental setup is shown in Figure 1. At 2 h before irradiation (after a 2 h oxaliplatin or 1.5 h irinotecan treatment) the complete medium was changed. For irinotecan alone and oxaliplatin alone experiments, medium without any drug was added. Alternatively, in order to test for the combined therapy regimes (oxaliplatin + 5-Fu and irinotecan + 5-Fu), medium containing 2µM 5-Fu was added at this time point. Since 5-Fu was standard of care for rectal cancer, cells treated with 5-Fu alone served as a control group. Native cells served as a second control group. Following treatment, cells were allowed to grow under standard culture conditions for 14 days with a medium change on day 7. Finally, the colonies were fixed with 70% ethanol, stained with 1% crystal violet and counted manually. The numbers of colonies containing more than 50 cells were counted, and the surviving fractions of cells were calculated. Clonogenic fractions of treated cells were normalized to the plating efficiency of untreated controls. Each independent experiment was conducted in duplicates.

### 4.6. γH2AX Foci Assay

The DNA double-strand breaks (DSB) were detected using the immunofluorescence of γH2AX foci. Moreover, 1 × 10^4^ cells/mL were seeded on glass slides 48 h before radiation. After, different incubation times cells were treated with drugs at the same scheme as described above and irradiated with 6 Gy. Cells without drug and with or without radiation served as negative controls. The samples were fixed 24 h after irradiation and following incubation under normal cell culture conditions to allow repair of ionizing radiation-induced DNA-DSB. The cancer cells were fixed and permeabilized first. After blocking, the cells were labelled for 1 h with the monoclonal anti-phospho-histone H2AX antibody at a dilution of 1:100 (Millipore, Schwalbach, Germany). After washing, the cells were incubated with the second antibody (Alexa Fluor 495 goat anti-mouse IgG, Invitrogen, Karlsruhe, Germany) at a dilution of 1:2000. Finally, the cells were covered with DAPI/antifade (MP Biomedicals, Eschwege, Germany) to stain the DNA. The number of foci was counted using an Eclipse TE300 inverted microscope (Nikon, Tokyo, Japan) with a magnification of 1000 diameters. Fifty cells were scored per glass slide. Each approach including controls was determined in duplicates; three independent experiments were performed.

### 4.7. Metabolic Assay

For a metabolic assay, 1 × 10^4^ cells per well were plated into 96-well plates. 24 h after cell seeding, drugs were applicated as described before (colony-forming assay), and 4 h later (28 h after seeding), cells were irradiated with 6 Gy or not (control, 0 Gy). At 1 h, 24 h and 48 h after irradiation, the complete medium was changed and test substance (XTT) of the EZ4U Cell Proliferation Assay (Biomedica Medizinprodukte GmbH & Co KG, Wien, Austria) was added and left for 2.5 h to detect metabolically formed formazan crystals. Absorbance was measured using an ELISA reader (anthos zenyth 340 r, Krefeld, Germany) at a wavelength of 492 nm and a reference wavelength of 620 nm. Each reaction was performed in eight replicates.

### 4.8. BrdU Assay

As a measurement of cell proliferation, a BrdU-assay (Roche Diagnostics, Mannheim, Germany) was performed. Therefore, 2 × 10^3^ cells per well were cultured in 96-well plates for 24 h and then treated with the drugs according to a specific time schedule as described before (colony-forming assay). Eight wells per assay were utilized for each reaction. BrdU was added 26 h after seeding at time point of medium change, and cells were irradiated 2 h later (28 h after seeding). At 1 h, 24 h or 48 h after irradiation, the assay’s specific instructions were followed for analysis. The ELISA-Reader (anthos zenyth 340 r, Krefeld, Germany) was used at a wavelength of 450 nm and a reference wavelength of 690 nm.

### 4.9. Statistical Analysis

Calculations were performed on the mean of at least three independent experiments. Data for radiosensibility experiments were pooled from at least three independent experiments with duplicate flasks in each experiment. Means are presented as the surviving fraction calculated from the plating efficiencies with respect to the corresponding controls. The survival curves were described as a linear–quadratic correlation. Error bars indicate the standard deviation of the mean for at least three independent experiments in the figures. The significance of treatments with various single drugs or drug combinations was determined with Student’s t-test. *p*-values are from two-sided tests. A significant difference was presumed when *p* < 0.05.

## Data Availability

The data presented in this study are available on request from the corresponding author.

## Abbreviations

5-FU: 5-Fluorouracil; CAPEOX: capecitabine, oxaliplatin; DMEM: Dulbecco’s modified Eagle medium; DSB: double-strand breaks; FBS: fetal bovine serum; FOLFOX: folinic acid [leucovorin], 5-fluorouracil, oxaliplatin; FOLFIRI: leucovorin, 5-fluorouracil, irinotecan; PBS: phosphate buffered saline; SN-38: 7-ethyl-10-hydroxycamptothecin, an active metabolite of irinotecan; TNT: total neoadjuvant therapy.
